# New Drugs, Appliances, and Things Medical

**Published:** 1890-04-19

**Authors:** 


### NEW DRUGS, APPLIANCES, AND THINGS MEDICAL.

[All preparations, appliances, novelties, etc., of which a notice is desired, should be sent to The Editor, The Lodge, Porchester Square, W.]

### DANIELSON AND CO.'S CLINICAL FIGURES AND CHARTS.

The above firm have sent us a variety of their productions from their warehouses at 52, Beaumont Street, W. We have examined them, and think that they are alike admirable in their idea and correct in their anatomy. They comprise, externally, outlines of the head, thorax, abdomen, and limbs; and internally, all the important regions of the body. Besides these, there are no less than 16 diagrams of deformities. The specialists in the eye, nose, ear, &c., have their wants attended to. The obstetrician and gynœcologist can make graphic representations of their cases; and finally, all the important bones and joints have plates assigned to them. Armed with these figures and outlines no man having an interesting case need despair of making a permanent note of its nature, as he has only to sketch on the outline the special features of the disease of his patient, and from that time has an accurate representation of the state of his patient at the date of making the note. There are also an admirable series of brain and spinal cord section outlines suggested by Dr. Ferrier. We note that all the different forms are gummed for insertion into the case-book. Besides these, the manufacturers publish useful temperature charts (three weeks), forms of instruction to patient and nurse, case sheets for case taking, and a very good "Perfect" clinical chart. The latter we have used with comfort for some time. A portfolio to hold case sheets, with alphabetical compartments, is an ingenious idea for arranging one's cases for permanent record, and seems likely to be of great use to those who intend to publish a series of cases of different natures, as the accounts can all be arranged under their several headings with great ease. We think Messrs. Danielson have made a distinct advance in these subjects, and wish them all success.

### DEE OIL COMPANY'S PREPARATIONS.

We have received from the above company samples of their manufacture. These are stated to be all produced by filtration processes and with no purifications over acid. On trying them with the tests required by the Pharmacopæa, we find that they answer to them all. We can confidently recommend them as they are pure and well manufactured varieties of parafinum molle. Amongst the varieties sent we notice the "Oleum Delinæ," a good specimen of the blue oil type; this is recommended as a liniment and an application to certain forms of eczema. Also a specimen of yellow petroleum jelly, "salvo petrolia," and finally, a neatly put up tube of white semi-solid petroleum, "edible salvo petrolia." This has been used with success for sore throats and other conditions of the upper air passages requiring an emollient application.

### TAYLOR'S SUPER A1 FLAX LINT.

This is an excellent specimen of lint, manufactured from flax fibre. It tears both ways and is decidedly strong. We would recommend the maker to also prepare it with some anti-septic, as nowadays lint is not much used unless in a state to satisfy the surgeon that it contains no germs. Put up with perchloride of mercury or boracic acid, it would leave nothing to be desired as its texture is admirable.

